# Maternal incarceration, child protection, and infant mortality: a descriptive study of infant children of women prisoners in Western Australia

**DOI:** 10.1186/s40352-018-0060-y

**Published:** 2018-01-15

**Authors:** Caitlin McMillen Dowell, Gloria C. Mejia, David B. Preen, Leonie Segal

**Affiliations:** 1 Health Economics and Social Policy Group, Centre for Population Health Research, Sansom Institute, School of Health Sciences, University of South Australia, GPO Box 2471, Adelaide, SA 5001 Australia; 2 Centre for Health Services Research, School of Population and Global Health, University of Western Australia, Crawley, WA Australia

**Keywords:** Women, Children, Prisoners, Infant mortality, Child protection, Vulnerable populations

## Abstract

**Background:**

There are no population statistics collected on a routine basis on the children of prisoners in Australia. Accordingly, their potential vulnerability to adverse outcomes remains unclear. This study draws on linked administrative data to describe the exposure of children aged less than 2 years to maternal imprisonment in Western Australia, their contact with child protection services, and infant mortality rates.

**Results:**

In Western Australia, 36.5 per 1000 Indigenous (*n* = 804) and 1.3 per 1000 non-Indigenous (*n* = 395) children born between 2001 and 2011 had mothers imprisoned after birth to age 2 years. One-third of infants’ mothers had multiple imprisonments (maximum of 11). Nearly half (46%) of prison stays were for ≤2 weeks, 12% were between 2 and 4 weeks, 14% were for 1–3 months, and 28% were longer than three months. Additionally, 17.4 per 1000 Indigenous (*n* = 383) and 0.5 per 1000 non-Indigenous (*n* = 150) children had mothers imprisoned during pregnancy. Half of the children with a history of maternal incarceration in pregnancy to age 2 years came into contact with child protection services by their second birthday, with 31% of Indigenous and 35% of non-Indigenous children entering out-of-home care. Rates of placement in care were significantly higher for Indigenous children (Relative Risk (RR) 27.30; 95%CI 19.19 to 38.84; *p* < .001) and for non-Indigenous children (RR 110.10; 95%CI 61.70 to 196.49; *p* < .001) with a history of maternal imprisonment compared to children of mothers with no corrections record. Infant mortality for children whose mothers were imprisoned up to 5 years before birth or within their first year after birth was higher than for children of mothers with no corrections record for both Indigenous (RR 2.36; 95%CI 1.41 to 3.95; *p* = .001) and non-Indigenous children (RR 2.28; 95%CI 0.75 to 6.97; *p* = .147).

**Conclusions:**

This study highlights the particular vulnerability of children whose mothers have been incarcerated and the importance of considering their needs within corrective services policies and procedures. Prison may present an opportunity to identify and work with vulnerable families to help improve outcomes for children as well as mothers.

## Background

Children of prisoners are highly vulnerable. Prisoners experience elevated rates of adversity including mental illness, domestic violence, substance use disorders, poverty, homelessness, racism and discrimination (Abbott, Magin, & Hu, 2016; Department of Corrective Services, 2009; Friestad, Ase-Bente, & Kjelsberg, 2012). These adverse experiences within a family environment are known to increase the risk of poor outcomes for children, particularly in the early years of infancy (Brown, Anda, Tiemeier, Felitti, Edwards, Croft, & Giles, 2009). Studies have found children with incarcerated parents experience a range of issues such as poor developmental outcomes, behavioural problems, educational difficulties, and increased mortality (Aaron, & Dallaire, 2010; Byrne, Goshin, & Joestl, 2010; Dallaire, Zeman, & Thrash, 2015; Rud, Van Klaveren, Groot, & Maassen van den Brink, 2014; Wildeman, Andersen, Lee, & Karlson, 2014). Much of the literature on children of prisoners has been conducted within the United States, United Kingdom and Europe (Murray, Bijleveld, Farrington, & Loeber, 2014). There is comparatively less known about the experience of children in other countries, including Australia. There is also relatively little evidence available on children of prisoners at a population level.

Within Australia, and beyond, there is no routine reporting of the prevalence of children affected by parental incarceration, nor their health or welfare (Dennison, Stewart, & Freiberg, 2013). Infant mortality is the most significant adverse outcome that can be experienced and is a strong marker of disadvantage and represents a measure of the health of a population (Reidpath, & Allotey, 2003). There is a lack of information on infant mortality outcomes for women prisoners, as distinct from imprisoned fathers, due to their relatively smaller numbers (Wildeman, 2012).

Child protection system contact is also an important measure of child welfare. Children with a history of placement in out-of-home care are at increased risk for a range of ongoing difficulties including mental illness, behavioural problems and poor school performance (Maclean, Taylor, & O’Donnell, 2016; Osborn, Delfabbro, & Barber, 2008). In addition, information on the characteristics of children’s exposure to parental incarceration is not readily available. There is no reporting on such characteristics as the duration and frequency of parental prison stays, or on the classification of parental offences. This information is important for establishing the health status and service needs of children of prisoners, and to inform future research priorities.

In the absence of formal population surveillance, data linkage provides a means for accurately describing the prevalence and characteristics of children of prisoners. Data linkage connects information relating to individuals within defined populations across multiple collections of data which are routinely recorded by government services and departments for primarily administrative purposes, such as hospital admission data or records of births, deaths and marriages (Holman, Bass, Rouse, & Hobbs, 1999). Data linkage can provide retrospective coverage of entire populations of interest, while maintaining the confidentiality of individuals, making it highly suitable for investigating marginalised groups such as the prisoner population, and rare outcomes such as infant mortality.

This study draws on linked data to provide key descriptive statistics for the population of infant children of women prisoners in Western Australia. Infants are a priority population given their vulnerability and unique developmental considerations. Infants have an absolute reliance on caregivers for their basic needs, lack communication skills, and have less structured contact with community services, such as schooling, where at-risk individuals may be identified. Experiences of adversity during pregnancy and infancy have significant and long-term consequences for child health and development. Exposure of the fetus during pregnancy or of the infant after birth to factors such as poor nutrition and maternal alcohol and drug abuse, domestic violence, and maternal stress has been shown to result in poor health outcomes including low birth weight, infant mortality and developmental delay (McMillen & Robinson 2005; Heindel, & Vandenberg, 2016; Latendresse et al., 2015). Women prisoners have increased risk of poor pregnancy outcomes including low birth weight infants when compared to women in the general community, as well an increased risk of disrupted attachment with their infants (Knight, & Plugge, 2005; Poehlmann, 2005). Despite their vulnerability, it is not known with any certainty how many Australian children experience the imprisonment of their mother in pregnancy or infancy, or the degree to which infants with a history of maternal imprisonment experience higher rates of mortality or child protection contact than infants in the general community.

There are also limited data on the infant children of Australian Indigenous women prisoners, despite their overrepresentation in the prison population. In Western Australia, for example, Indigenous peoples represent 4% of the general population but 46% of the female prison population (Australian Bureau of Statistics (ABS), 2016; ABS, 2011). Indigenous children are also overrepresented in the Australian child protection system. Similarly, Indigenous peoples in Australia experience poorer pregnancy outcomes compared with non-Indigenous mothers, and while infant mortality rates have improved across time for both Indigenous and non-Indigenous populations, a racial disparity remains (Council of Australian Governments (COAG) 2016). This reflects the high levels of disadvantage and discrimination experienced by Indigenous peoples in Australia stemming from the historical and continuing impacts of colonisation, such as land dispossession, racism, and the forced removal of children from their families (COAG, 2009).

Accordingly this study will provide a description of the population of infant children of Western Australian women prisoners including details of the characteristics of exposure of infant children (aged less than 2 years) to maternal imprisonment, as well as measuring child protection system contact and infant mortality rates for the population.

## Methods

### Aims

This study draws on whole-population linked administrative data to examine maternal incarceration exposure, child protection system contact, and mortality rates for infant children of Western Australian women prisoners.

Aim 1: To describe the features of maternal incarceration exposure including the proportion of women prisoners who are pregnant; the prevalence of children exposed to maternal incarceration between birth and before age-2; and offence type and length and frequency of maternal imprisonments for children between birth and before age-2.

Aim 2: To describe contact with the child protection system before 2 years of age for children whose mothers have a record of imprisonment during pregnancy or before the child’s second birthday and for children whose mothers have no corrections record.

Aim 3: To determine infant mortality rates for children of women with a history of imprisonment 5 years before birth or within their first year after birth and for children whose mother had no contact with corrective services.

### Data sources

Data were obtained through the Western Australian Data Linkage System (WADLS). The WADLS uses highly accurate computerised probabilistic matching with clerical review to create linkages between administrative data collections across a range of Western Australian government departments and services (Holman et al., 1999). For the present study, records were extracted from the Midwives Notifications System, Birth Registrations, Death Registrations, Department of Justice, and Department of Communities: Child Protection and Family Support data collections. These are all State-wide and statutory data collections with good coverage of the Western Australian population.

The Birth Registration and Midwives Notifications System data provides social and demographic characteristics of mothers and children at time of birth. Death data includes all deaths registered in Western Australia. The Department of Justice data collections include all custodial records for offenders held in Western Australian prisoners or under supervision of the Department on community-based correctional orders. The Department of Communities: Child Protection and Family Support data includes all reports of concerns for child welfare made to the child protection system and the details of investigations, protection applications and orders as well as placements in out-of-home care.

### Study population

The study population was drawn from a retrospective longitudinal cohort study of all children born in Western Australia between 1985 and 2011 whose biological mother was imprisoned at least once between their date of birth and 18th-birthday, identified using the Midwives Notification System and Registry of Births in conjunction with linked Department of Justice prison data. For a description of the full cohort see Dowell, Preen, and Segal (2017). The cohort study included a randomly sampled comparison group of children whose mother had no record of imprisonment from their birth to their 18th-birthday, which was identified through the same data sources as the cohort and matched 3:1 to cohort children on Indigenous status, age and gender.

The present study focused on a subset of children within the full cohort born from 2001 onwards. This time period was chosen due to substantial changes in the annual prevalence of children affected by maternal incarceration in Western Australia in the 1990s (Dowell et al., 2017). To address the three study aims, we have conducted three separate analyses all of which have slightly different observation periods, and hence different study populations, as summarised in Table 1. All analyses were stratified by Indigenous status given differences between Australian Indigenous and non-Indigenous populations in terms of their demographic characteristics, health status, sentence type and unique cultural contexts.

**Table 1:** Observation periods for key analyses

Aim/Analyses	Maternal imprisonment observation period	Child birth years	Data observation years	Prison group n	Comparison group n
1. Maternal incarceration exposure					
In pregnancy	Pregnancy	2001–2011	2000–2011	533	–
In infancy	Birth to age < 2	2001–2011	2001–2013	1199	–
2. Child protection system contact					
All children	Pregnancy to age < 2	2001–2011	2000–2017	1383	6714
3. Infant mortality rates					
All children	5-years pre-birth to age < 1	2001–2011	1996–2012	2180	6714
Indigenous children (Fig. 2)	5-years pre-birth to age < 1	1992–2011	1987–2012	2818	7190

To address aim 1, maternal incarceration exposure was described for pregnancy and after birth to before age 2-years separately, because of the different implications of imprisonment for unborn and infant children. The study population included children born 2001–2011 whose mother was imprisoned in pregnancy between 2000 and 2011 (*n* = 533) and children whose mother was imprisoned from birth to before age-2 between 2001 and 2013 (*n* = 1199). There were 349 children within both groups who were exposed to maternal imprisonment in pregnancy and after birth before age-2. Mothers could have multiple children who met the inclusion criteria.

To address aim 2, child protection system contact was described from the start of pregnancy through to before age-2, as it is possible for child protection notifications to be raised for unborn children in Western Australia.

To address aim 3, we studied children born to mothers with a history of imprisonment 5-years before birth to age-1 (*n* = 2180) as infant mortality is a rare outcome in Australia and there were not sufficient numbers of infant deaths to limit analyses to children of mothers imprisoned in pregnancy and their first year. Accordingly, the analyses explore the population characteristics and mortality outcomes of children whose mothers have a history of imprisonment. Not all children in our defined at-risk group will be directly exposed to maternal imprisonment in utero or after birth. Infant mortality rates for Indigenous children born from 1992 onwards were calculated in order to illustrate the change in rates over time. A similar calculation was not possible for non-Indigenous children, due to small numbers. Western Australian birth data for population denominators are only available by Indigenous status from 1992 onwards (ABS, 2015).

The comparison groups were also stratified by Indigenous status and limited to children whose mothers never had a record in any Department of Justice data collection (including for community-based correctional orders) at any time during the period from 1985 to 2014. These groups were selected to describe the difference in child protection system contact and infant mortality between infants of women prisoners and infants within a general community sample with no maternal corrections history.

### Analyses

#### Maternal incarceration exposure

The proportion of women prisoners who are pregnant was calculated from the total number of Western Australian women prisoners using Australian Prisoner Census data (ABS, 2016). As the Prisoner Census records the prison population on a single day (30 June) each year, the numbers of pregnant women in custody on 30 June annually were identified from the study data using mothers’ prison reception and discharge dates and children’s birthdates. Pregnancy start date was calculated as being nine months before child birth-month, as child birthdate was provided to month of birth only and gestational age was not available.

The prevalence of children whose mother was imprisoned during their infancy (birth to age 2-years) was calculated for Indigenous and non-Indigenous children born in Western Australia across the period of 2001 to 2011 using population birth data from the ABS (ABS, 2015). The number, length and nature of offence for maternal imprisonments during infancy, were described using child birth and death dates, mother prison reception and discharge dates, sentence type, and major offence type using the Australian and New Zealand Standard Offence Classification (Pink, 2011). Maternal imprisonments included both juvenile and adult sentenced offenders and remandees held in Western Australian prisons.

#### Child protection system contact

Child protection system contact was defined as any record within the Western Australian Department of Communities: Child Protection and Family Support database for reports made to the Department concerning a child’s welfare, applications or orders for a child’s protection, and periods of out-of-home care (including for respite care as well as child maltreatment).

The proportion and rate (per 1000) of children who had any child protection contact (including out-of-home care) was calculated for children whose mother had a record of imprisonment during pregnancy and/or from birth to age 2-years, and for those whose mother never had a corrections record. The proportion and rate of children who were placed in out-of-home care was also calculated.

#### Infant mortality

Infant mortality rate is commonly defined as “the number of deaths under one year of age occurring among the live births in a given geographical area during a given year, per 1000 live births occurring among the population of the given geographical area during the same year” (Organisation for Economic Co-operation and Development (OECD) 2006). All children within the study population were live-born. Infant mortality rates (per 1000) across the period 2001–2011 were calculated for children with a history of maternal imprisonment and children of mothers with no corrective services history stratified by Indigenous status.

Infant mortality rates were calculated for Indigenous children born from 1992 to 2011 using 6-year moving averages (e.g. 1992–1997, 1993–1998, etc.) and presented for children of mothers with an incarceration history, children of mothers with no history of corrective services contact, and for all Western Australian Indigenous children, the latter using ABS data.

### Indigenous status

Indigenous status for children and mothers in the study populations was provided through the Derived Indigenous Status Flag variable generated by the WADLS using best-practice algorithms, which assess individuals’ Indigenous status across multiple data collections to enhance accuracy (Christensen et al., 2014).

### Offence type

Major offence type is recorded in the Department of Justice data collections using the Australian and New Zealand Standard Offence Classification, which contains 16 Divisions classified according to the offence aim, use of violence, victim, seriousness and intent (Pink, 2011). For the present study, offence type were described in six categories: Offences against the person (Divisions 1–5); Theft and related (Divisions 6–8); Fraud and related (Division 9 and bribery offences); Drug (Division 10 and licit drug offences); Public order (Divisions 11–13 and 16); Traffic and related (Division 14, and driving under the influence of alcohol or other substance, dangerous or negligent driving offences); and Breach of justice procedures (Division 15).

### Socioeconomic status and geographical remoteness

The Socio-economic Indexes for Areas Index of Relative Socio-economic Disadvantage (ABS, 2013) and the Accessibility/Remoteness Index of Australia (ABS, 2014) were used to describe infant’s socioeconomic status and geographical remoteness of their place of residence at time of birth.

### Statistical analysis

All statistical analyses were performed in Stata version 14.0 and stratified by Indigenous status. The analyses were undertaken to establish whether the study populations differed in terms of their key characteristics. Pearson’s chi-square was used to assess the difference between study sub-populations’ basic demographic characteristics including sex, socioeconomic status, and geographical remoteness. A log-binomial regression model was applied to the child protection and infant mortality data to obtain risk ratios for comparing children of women prisoners with children whose mothers had no corrections record (Knol, Le Cessie, Algra, Vandenbroucke, & Groenwold, 2011; McNutt, Wu, Xue, & Hafner, 2003; Robbins, Chao, & Fonseca, 2002).

## Results

### Descriptive characteristics of the study sub-populations

Table 2 describes the basic demographic characteristics of the study populations. Within the Indigenous and non-Indigenous populations, there were no statistically significant differences (*p* > .05) between the two maternal imprisonment history subgroups across all characteristics. Likewise, within each of the Indigenous and non-Indigenous populations, there were no significant differences with respect to the sex distributions of the groups of infants with maternal imprisonment histories to those with no maternal corrections record. In both the Indigenous and non-Indigenous populations, infants with a history of maternal imprisonment had different distributions of socioeconomic status and geographical remoteness in comparison to infants with no maternal corrections history.

**Table 2:** Descriptive characteristics of the study sub-populations, by Indigenous status

	Indigenous			Non-Indigenous		
	Maternal imprisonment		No maternal corrections	Maternal imprisonment		No maternal corrections
	Pregnancy to <2 yrs	5 yrs. before birth- < 1 yr		Pregnancy to <2 yrs	5 yrs. before birth- < 1 yr	
	%	%	%	%	%	%
Sex						
Male	50.2	50.4	52.4	50.8	51.3	48.0
Female	49.8	49.6	47.6	49.2	48.7	52.0
Socioeconomic status						
Most disadvantage, 0–20%	58.7	58.5	60.0	42.0	42.9	20.5
21–40%	21.7	22.4	21.3	22.7	21.3	23.2
41–60%	11.8	11.8	8.9	18.1	16.5	16.3
61–80%	4.6	4.7	7.0	9.6	11.6	22.6
Least disadvantage, 81–100%	3.2	2.6	2.8	7.6	7.7	17.3
Geographical remoteness						
Major cities	59.1	59.1	32.2	81.9	81.5	70.6
Inner regional	4.5	5.5	7.4	11.5	11.2	12.3
Outer regional	13.4	15.3	16.9	5.3	5.7	10.1
Remote^a^	11.0	9.6	20.4	1.4	1.6	7.0
Very remote^a^	12.0	10.5	23.2			
Infants (n)	944	1283	2923	439	511	3791

Notes

^a^‘Remote’ and ‘Very remote’ categories combined for non-Indigenous due to cell counts of *n* < 5

### Maternal incarceration exposure

#### Pregnancy

Between 2001 and 2011, 5.7% of Indigenous and 4.0% of non-Indigenous Western Australian female prisoners were pregnant while imprisoned. Over the same period 383 Indigenous (17.40 per 1000) and 150 non-Indigenous (0.49 per 1000) children were born to mothers who were imprisoned at least once while pregnant with them.

#### Birth to two-years

Table 3 provides a summary of children’s exposure to maternal incarceration in infancy. In total, 804 Indigenous and 395 non-Indigenous children had a mother imprisoned at least once between their birth and their second birthday, equating to 36.52 per 1000 Indigenous and 1.29 per 1000 non-Indigenous children born in Western Australia between 2001 and 2011. Of these children, 30% of Indigenous and 27% of non-Indigenous, also had their mother imprisoned while pregnant with them.

**Table 3:** Maternal incarceration exposure in infancy (birth to age 2-years) for Western Australian children born 2001–2011

	Indigenous		Non-Indigenous	
Characteristics	n	%	n	%
Child experience of maternal incarceration				
Mother imprisoned in infancy (not pregnancy)	561	69.8	289	73.2
Mother imprisoned in infancy and pregnancy	243	30.2	106	26.8
Number of maternal imprisonments in infancy, per child				
1	509	63.3	297	75.2
2	170	21.1	65	16.5
3	66	8.2	20	5.1
4+	59	7.3	13	3.3
Percentage of child’s infancy mother was imprisoned				
0–5%	428	53.2	202	51.1
6–25%	203	25.2	104	26.3
26–50%	114	14.2	70	17.7
51–75%	47	5.8	14	3.5
76–100%	12	1.5	5	1.3
Length of maternal prison stays^a^				
Up to 2 weeks / 0–14 days	620	46.7	234	43.0
2–4 weeks / 15–30 days	154	11.6	63	11.6
1–3 months / 31–90 days	186	14.0	80	14.7
3–6 months / 91–180 days	138	10.4	61	11.2
6–12 months / 181–365 days	147	11.1	68	12.5
Greater than 1 year / 366+ days	82	6.2	38	7.0
Sentence type^a^				
Unsentenced – remand	683	51.5	265	48.7
Sentenced – default of fine	250	18.8	91	16.7
Sentenced – other	394	29.7	188	34.6
Offence type^ab^				
Against the person	361	29.3	87	16.6
Theft and related	389	31.6	162	30.9
Fraud and related	39	3.2	59	11.3
Drug offences	12	1.0	45	8.6
Public order	45	3.7	12	2.3
Traffic and related	173	14.0	59	11.3
Breach of justice procedures	213	17.3	100	19.1

^a^1871 maternal prison records in infancy (1327 Indigenous and 544 non-Indigenous)

^b^115 prison records missing offence type (95 Indigenous and 20 non-Indigenous)

For most children exposed to maternal incarceration between birth and age 2-years, their mothers were imprisoned just once (63% Indigenous and 75% non-Indigenous children). The mean (SD) length of incarceration was 97 (184) days but the distribution was highly skewed, with half of all mothers’ prison stays being for periods less than three-weeks with a median (IQR) of 19 (4–110) days.

The level of exposure varied considerably across the population of children exposed to maternal incarceration with 37% of Indigenous and 25% of non-Indigenous children experiencing multiple (up to 11) periods of maternal incarceration during infancy. For 22% of Indigenous and 23% of non-Indigenous children, their mother was imprisoned in total for more than a quarter of their life to age 2-years.

As shown in Table 3, mothers of children aged less than 2-years are more frequently imprisoned for non-violent offences related to theft, breach of justice procedures, and traffic offences. Offences against a person (e.g., acts intended to cause injury) accounted for 29% of Indigenous and 17% of non-Indigenous mothers’ imprisonments.

### Child protection system contact

As shown in the Table 4, a considerably higher proportion of children had contact with the Western Australian child protection system by their second birthday if their mother was imprisoned during pregnancy or infancy, compared with children whose mother never had any involvement with corrective services.

**Table 4:** Child protection system contact before and after age 2 years, by maternal imprisonment history

	Indigenous children				Non-Indigenous children			
	Maternal imprisonment		No corrections record		Maternal imprisonment		No corrections record	
Child protection contact	n	%	n	%	n	%	n	%
Child protection contact by age-2	537	56.9	208	7.1	243	55.4	74	2.0
*Out-of-home care by age-2*	*291*	*30.8*	*33*	*1.1*	*153*	*34.9*	*12*	*0.3*
No child protection contact by age-2	407	43.1	2715	92.9	196	44.6	3717	98.0
*Child protection contact age-2+*	*213*	*22.6*	*527*	*18.0*	*84*	*19.1*	*145*	*3.8*
*Out-of-home care age-2+*	*78*	*8.3*	*67*	*2.3*	*30*	*6.8*	*20*	*0.5*
Total	944	100	2923	100	439	100	3791	100

For children born between 2001 and 2011 with a history of maternal imprisonment in pregnancy or infancy, 537 (568.9 per 1000) Indigenous and 243 (553.5 per 1000) non-Indigenous children came into contact with the child protection system by their second birthday. Of those children, 291 (31%) Indigenous and 153 (35%) non-Indigenous children were removed and placed in out of home care by age 2.

In comparison to children whose mothers had no corrections involvement, children of mothers with a prison record in pregnancy or infancy were 8-times (RR 7.99; 95%CI 6.93 to 9.22, *p* < .001) and 28-times (RR 28.36; 95%CI 22.29 to 36.08, *p* < .001) more likely to have contact with the child protection system by age 2 years for Indigenous and non-Indigenous children respectively. Rates of placement in out-of-home care by age-2 were 27-times (RR 27.30; 95%CI 19.19 to 38.84, *p* < .001) greater for Indigenous and 110-times (RR 110.10; 95%CI 61.70 to 196.49, *p* < .001) greater for non-Indigenous children whose mothers had a prison history during pregnancy to age 2-years compared to children whose mothers had no contact with corrections.

For the children of mothers with a record of imprisonment in pregnancy or infancy who had no child protection contact by age 2-years, a further 23% Indigenous and 19% non-Indigenous had child protection contact after their second birthday. A further 8% of Indigenous and 7% of non-Indigenous children had spent time in out-of-home care. This compared with 18% Indigenous (2% out-of-home care) and 4% non-Indigenous (0.5% out-of-home care) for children of mothers with no record of corrections contact.

By the end of the observation period in 2017, 21% of Indigenous and 26% non-Indigenous children with maternal imprisonment history in pregnancy or infancy had no child protection contact. This compares with 75% of Indigenous and 94% of non-Indigenous children of mothers with no corrections involvement.

### Infant mortality

Across 2001–2011, Western Australian children whose mothers had a history of imprisonment prior to their birth (up to five years prior) or within their first year of life had an infant mortality rate of 22.6 deaths per 1000 Indigenous births and 7.8 deaths per 1000 non-Indigenous births. Infant mortality rates were higher for children of women prisoners compared to children of mothers who had no contact with corrective services by 2.36-times (95%CI 1.41 to 3.95, *p* = .001) for Indigenous and 2.28-times (95%CI 0.75 to 6.97, *p* = .147) for non-Indigenous children.

**Fig. 1:**
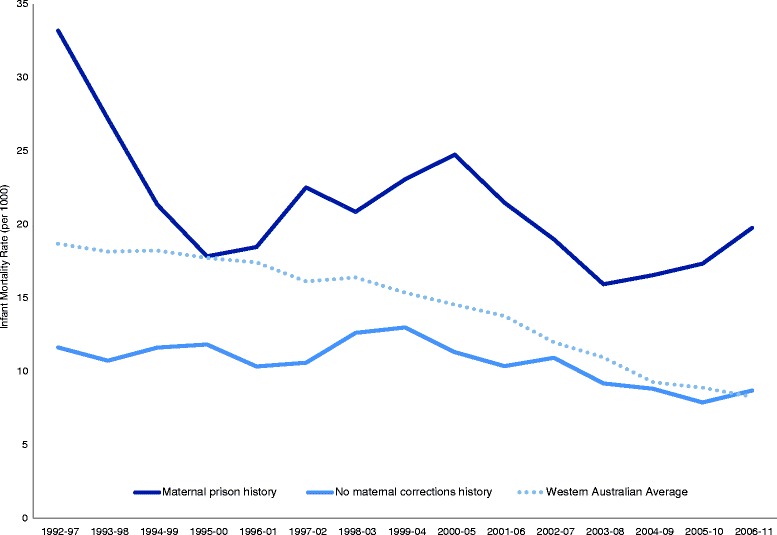
Indigenous infant mortality rate by maternal corrections history, Western Australian children born 1992–2011 (6-year average)

Figure 1 shows the temporal change and difference in infant mortality rates between Indigenous children whose mother had a history of imprisonment and those whose mother had no corrections record, as well as the Western Australian average for Indigenous children. Between 1992 and 97 and 2006–11, infant mortality rates for Western Australian Indigenous children reduced by 56% overall and by 40% for children with maternal prison history, while there was less change in rates for children with no maternal corrections history with a 25% reduction over the period.

## Discussion

A major finding of the study is that 1 in 3 children whose mothers were imprisoned during pregnancy or their first two years of life, were placed in out-of-home care by their second birthday. In contrast only 1 in 100 Indigenous and 1 in 300 non-Indigenous children whose mothers had never been in contact with corrective services were placed in out of home care by the same age. While parental imprisonment has been recognised as a factor that contributes to infants and children being placed in care (Delfabbro, Borgas, Rogers, Jeffrey, & Wilson, 2009), the extent to which the population of women prisoners have children placed in care has not been previously quantified in Australia.

In our study, half of all imprisonments were for unsentenced mothers being held in custody on remand. In such circumstances it is possible mothers may not have time to arrange care for their children following their arrest (Healy, Foley, & Walsh, 2001; Dallaire, 2007). Nearly half of all imprisonments of mothers whose children were aged less than 2 years were for two-weeks or less. This raises the question of if and how the length of a mother’s prison stay impacts on the likelihood of child protection contact. Prisoners held on remand or for short periods would be difficult to capture in prospective research and so have not featured much in prior studies. The assumption is often that longer periods of imprisonment are potentially more harmful for young children, as in theory they are more likely to lead to disrupted attachment. But it is possible that shorter imprisonments, and unplanned detention in the case of remandees, create a greater disruption for already vulnerable families. Questions have been raised about the utility of short-term imprisonment in general (Trevena, & Weatherburn, 2015; Wermink, Blokland, Nieuwbeerta, Nagin, & Tollenaar, 2010). The data reported in this study highlight the importance of considering the value of very short-term maternal imprisonments. Such periods are unlikely to allow for any participation in rehabilitative programs and may increase the likelihood of children coming into contact with the child protection system, although whether such contact is helpful or harmful to the child is not known.

One in six of the maternal imprisonments in the cohort were for non-payment of fines. While nearly a third of maternal imprisonments for the Indigenous population were for violent offences against the person, a significant number of imprisonments for both Indigenous and non-Indigenous mothers were for non-violent crimes and fine default. There was legislative action taken to reduce imprisonments for non-payment of fines in Western Australia in the mid-1990s with the enactment of the *Fines, Penalties and Infringement Notices Enforcement Act* 1994 (WA). This appeared to lead to a significant reduction in maternal imprisonments from 1994 to 1996 (Dowell et al. 2017), although the current study demonstrates that default of fine remains a common reason for imprisonment of Western Australian women prisoners with young children. The Western Australian legislation has since been described as having “a discriminatory and disproportionate effect, leading to the over-representation of Aboriginal and Torres Strait Islander peoples, poor people and vulnerable people, in the Western Australian prison system” (Law Society of Western Australia, 2016). Female prisoners are more frequently imprisoned for default of fine than male prisoners in Western Australia (Morgan, 2016). Three-quarters of women imprisoned for fine default in Western Australia are not employed, in comparison to 10% of males (Morgan, 2016). Socioeconomic factors including homelessness can also lead to prisoners being detained on remand in circumstances where they would otherwise be released before trial (Ayres, Heggie, & de Almeida Neto, 2010). While it is well established that women prisoners experience high levels of socioeconomic disadvantage, the policy discussion around short term, remand, and fine default imprisonments has not extended to consider the potential implications of these for children with incarcerated mothers.

We found that for children aged less than 2 years, rates of contact with the child protection system were almost identical for Indigenous and non-Indigenous children whose mothers were imprisoned during pregnancy or before their second birthday. However, twice the number of Indigenous compared to non-Indigenous children had their mother imprisoned between pregnancy and their second birthday. This is of concern given that less than 7% of Western Australian children under 4 years old are Indigenous (ABS, 2011). The over-representation of Indigenous children within the child protection system has been well established (Delfabbro, Hirte, Rogers, & Wilson, 2010). Our study findings raise the question of how much of the over-representation of young Indigenous children in the child protection system may be related to the over-representation of Indigenous women in the prison system. The removal of Indigenous children from families is a pertinent issue in Australia, with some commentators expressing concerns in the wake of the rise in numbers of Indigenous children in care (Lavarch, 2016).

It would be valuable to explore whether there is any association between the timing of maternal incarceration and child protection contact but this is beyond the scope of the present paper given the complexities we have reported of prison exposure in terms of frequency and duration, and taking account of similar complexities of the timing, frequency and nature of child protection system contacts, familial relationships and additional contributing factors. The magnitude of our findings suggest the interaction between maternal incarceration and exposure of children to the child protection system is significant. The relationship between maternal incarceration, child protection and juvenile justice system involvement is clearly an important area for future research.

Our study found that infant mortality rates for children of mothers with a history of imprisonment were over twice the rate for children of mothers with no corrections record. This equated to an additional 13.1 deaths per 1000 Indigenous births and 4.4 deaths per 1000 non-Indigenous births, for children whose mothers have a history of imprisonment. While we were unable to calculate rates for women imprisoned during pregnancy separately, the international literature on the birth outcomes of women imprisoned during pregnancy has found improved birth outcomes, including birth weight, for women imprisoned during pregnancy compared to women imprisoned at other times and worse birth outcomes compared to women who are never imprisoned (Knight, & Plugge, 2005). However, these findings were not replicated by the only Australian study to date by Walker, Hilder, Levy, and Sullivan (2014), which found similarly poor birth outcomes for women imprisoned while pregnant compared to women imprisoned at other times. The authors questioned whether this was in part due to the shorter imprisonment terms within the New South Wales female prison population compared to those in the United States (Walker et al., 2014). Our findings highlight the increased risk of infant mortality for children born to mothers with a history of imprisonment. Further research is needed, however, to establish whether the experience of maternal incarceration itself increases children’s infant mortality risk, or whether it is acting as a marker of extreme vulnerability.

### Limitations

The sampling methodology for the broader longitudinal study, from which the data were drawn, primarily identified children whose mothers had at least one record of imprisonment after their birth to their 18th-birthday. While it was possible to identify women who were imprisoned while pregnant, the sample did not include a full census of women imprisoned during pregnancy in Western Australia. Possible implications of this would be an underreporting of the proportion of Western Australian female prisoners who are pregnant, however, we found similar proportions of female prisoners who are imprisoned while pregnant as Australian-wide data (Australian Institute of Health and Welfare, 2014). Thus any error is likely to be small.

Data were not available on gestational age and the day of birth, accordingly the date of commencement of pregnancy was taken as nine-months before the first day of a child’s birth month. This may have included some mothers who were not yet pregnant at time of imprisonment, but were at least nine months away from giving birth. Research has shown, however, that the preconception period (approximately 6 months prior to conception) is also an important time when the experience of maternal stress during this period can impact on the health of the conceived child (Class, Khashan, Lichtenstein, Långström, & D’Onofrio, 2013).

## Conclusions

This is the first Australian study to report the extent of the population of infant children of women prisoners in contact with the child protection system. The results confirm that infant children of women prisoners are a highly vulnerable population who experience a significantly increased risk of engagement with the child protection system and placement in out-of-home care. These children also experience high rates of infant death compared to children of mothers with no corrective services history. The findings also demonstrate that many imprisoned mothers of young children are held on remand, for short periods of time and for non-violent crimes. This study highlights the importance of considering the heterogeneity of timing and frequency of imprisonment within the prison population on outcomes for families and children.

## Abbreviations

ABS: Australian Bureau of Statistics
COAG: Council of Australian Governments
OECD: Organisation for Economic Co-operation and Development
WADLS: Western Australian Data Linkage System
